# Supplementary material for: GA4GH Passport standard for digital identity and access permissions
Source: Cell Genom. 2021 Nov 10;1(2):100030. doi: 10.1016/j.xgen.2021.100030 (PMC8591913; doi:10.1016/j.xgen.2021.100030)
Supplement: Document S1. Controlled access grant example, GA4GH Passport overview, and webinars [file mmc1.pdf]

Cell Genomics, Volume *1*

Supplemental information

GA4GH Passport standard for digital identity and access permissions

Craig Voisin, Mikael Linden, Stephanie O.M. Dyke, Sarion R. Bowers, Pinar Alper, Maxmillian P. Barkley, David Bernick, Jianpeng Chao, Mélanie Courtot, Francis Jeanson, Melissa A. Konopko, Martin Kuba, Jonathan Lawson, Jaakko Leinonen, Stephanie Li, Vivian Ota Wang, Anthony A. Philippakis, Kathy Reinold, Gregory A. Rushton, J. Dylan Spalding, Juha Törnroos, Ilya Tulchinsky, Jaime M. Guidry Auvil, and Tommi H. Nyrönen

Supplemental Information for *GA4GH Passport standard for digital identity and access permissions*

Table of Contents:

Controlled Access Grant Example Page 1
GA4GH Passport Overview Page 3
Webinars Page 3

Controlled Access Grant Example

The example below illustrates how ControlledAccessGrants visa works.
A visa is a JSON Web Token (JWT, RFC 7519) which is electronically “signed” by the Visa issuer. Note that the JWT has been decoded and the header and signature stripped to improve human readability.

In this example, a Visa Issuer has delivered a visa to a Passport broker which has further exposed the visa to a Passport Clearinghouse from a standard OIDC userinfo endpoint, in exchange of a valid access token, as defined by the GA4GH AAI standard.


```
{
  "iss" : "https://ega.ebi.ac.uk:8053/ega-openid-connect-server/",
  "sub" : "EGAW1234567890",
  "ga4gh_visa_v1" : {
          "type" : "ControlledAccessGrants",
          "asserted" : 1578912709,
          "value" : "https://ega-archive.org/datasets/EGAD00001003338",
          "source" : "https://ega-archive.org/dacs/EGAC00001000620",
          "by" : "dac"
  },
  "exp" : 1592494992,
  "iat" : 1592491392,
  "jti" : "119b5384-3319-46d2-9cc3-9a5aa7db58aa"
}
```


The "ga4gh_visa_v1" JWT claim is specified by the GA4GH Passport Visa Format specification whereas the other common JWT claims are specified by the GA4GH AAI Profile Embedded Document Format specification.

The example below exposes the technical protocol flow (Figure 7) that can be used for accessing a Beacon, a data discovery service based on the related GA4GH standard. Other implementations may vary in their service

interface and still comply with the GA4GH Passport standard. In this architecture the authenticated data user interacts with the system via a web browser, a command line tool or a graphical user interface.

[Figure 7]

1. Redirect user to Broker’s /authorize endpoint

```
HTTP/1.1 302 Found
Location: https://login.elixir-czech.org/oidc/authorize
      ?response_type=code
      &scope=openid+ga4gh_passport_v1
      &client_id=<your-client-id>
      &state=<nonce>
```

2. Broker redirects user back to your registered callback address

```
HTTP/1.1 302 Found
Location: https://your.host.org/callback
    ?code=<authorisation-code>
    &state=<nonce>
```

3. Send authorisation-code to Broker’s /token REST API

```
POST https://login.elixir-czech.org/oidc/token HTTP/1.1
Content-Type: application/x-www-form-urlencoded
Authorization: Basic <your-client-id><client-secret>

grant_type=authorization_code
    &code=<authorisation-code>
    &redirect_uri=https://your.host.org/callback
```

4. Receive user’s Access token in response

```
HTTP/1.1 200 OK
Content-Type: application/json
{
"access_token": "<access-token>",
"expires_in": 3600,
“token_type”: “Bearer”,
“scope”: “openid ga4gh_passport_v1”,
"id_token": "<id-token>"
}
```

5. Attach the bearer token to a GA4GH API call

```
GET /query HTTP/1.1
 Host: beacon.example.com
 Authorization: Bearer <access-token>
```

Note: In step #5, this is the mechanism supported in the v1.0 specification however NIH is implementing new mechanisms and implementations that provide finer granularity of control.

6. Client receives a response from the GA4GH API call

```
HTTP/1.1 200 OK
Content-Type: application/json

{
"beaconId": "<id>",
"exists": true,
...
}
```

GA4GH Passport Overview

Where the GA4GH Passport standard fits in with other GA4GH standards to assist with discovery, access, and analysis of global datasets. The **GA4GH Passport Overview Video** may be found in the Key Resources Table.

Webinars

Webinar GA4GH Passports: Benefits of Integrating a Global Electronic ID for Accessing Biomedical Data (Part 1)

Video: https://youtu.be/I5Cu76NQyUY
Summary:
https://www.ga4gh.org/news/webinar-recap-ga4gh-passports-benefits-of-integrating-a-global-electronic-id-for-accessing-biomedical-data-part-1/

Webinar GA4GH Passports: Implementing GA4GH Passports and AAI: Technical Deep Dive (Part 2)

Video: https://youtu.be/K7HID5KAhz0
Summary:
https://www.ga4gh.org/news/webinar-recap-ga4gh-passports-implementing-ga4gh-passports-and-aai-technical-deep-dive-part-2/
